# Controlled temperature chain for vaccination in low- and middle-income countries: a realist evidence synthesis

**DOI:** 10.2471/BLT.21.287696

**Published:** 2022-06-22

**Authors:** Christopher P Seaman, Anna-Lea Kahn, Debra Kristensen, Robert Steinglass, Dijana Spasenoska, Nick Scott, Christopher Morgan

**Affiliations:** a Burnet Institute, 85 Commercial Road, Melbourne, Victoria 3004, Australia.; b Immunization, Vaccines and Biologicals Department, World Health Organization, Geneva, Switzerland.; c Bend, Oregon, United States of America (USA).; d Mars Hill, North Carolina, USA.; e Department of Social Policy, The London School of Economics and Political Science, London, England.; f Jhpiego, Johns Hopkins University affiliate, Baltimore, USA.

## Abstract

**Objective:**

To evaluate the evidence describing how the controlled temperature chain approach for vaccination could lead to improved equitable immunization coverage in low- and middle-income countries.

**Methods:**

We created a theory of change construct from the *Controlled temperature chain: strategic roadmap for priority vaccines 2017–2020,* containing four domains: (i) uptake and demand for the approach; (ii) compliance and safe use of the approach; (iii) programmatic efficiency gains from the approach; and (iv) improved equitable immunization coverage. To verify and improve the theory of change, we applied a realist review method to analyse published descriptions of controlled temperature chain or closely related experiences.

**Findings:**

We evaluated 34 articles, describing 22 unique controlled temperature chain or closely related experiences across four World Health Organization regions. We identified a strong demand for this approach among service delivery providers; however, generating an equal level of demand among policy-makers requires greater evidence on economic benefits and on vaccination coverage gains, and use case definitions. Consistent evidence supported safety of the approach when integrated into special vaccination programmes. Feasible training and supervision supported providers in complying with protocols. Time-savings were the main evidence for efficiency gains, while cost-saving data were minimal. Improved equitable coverage was reported where vaccine storage beyond the cold chain enabled access to hard-to-reach populations. No evidence indicated an inferior vaccine effectiveness nor increased adverse event rates for vaccines delivered under the approach.

**Conclusion:**

Synthesized evidence broadly supported the initial theory of change. Addressing evidence gaps on economic benefits and coverage gains may increase future uptake.

## Résumé

**Objectif:**

Évaluer les preuves exposant la manière dont la chaîne de température contrôlée pourrait constituer l'approche idéale pour garantir une couverture vaccinale plus équitable dans les pays à revenu faible et intermédiaire.

**Méthodes:**

Nous avons élaboré un concept basé sur la théorie du changement figurant dans *Chaîne de température contrôlée: feuille de route stratégique pour les vaccins prioritaires 2017–2020,* et portant sur quatre domaines: (i) demande et adhésion à l'approche; (ii) respect et sécurité d'utilisation de l'approche; (iii) avantages de l'approche au niveau de l'efficacité des programmes; et enfin, (iv) couverture vaccinale plus équitable. Afin de vérifier et d'affiner la théorie du changement, nous avons employé une méthode de synthèse réaliste destinée à analyser les descriptions publiées concernant la chaîne de température contrôlée ou toute autre expérience étroitement liée au sujet.

**Résultats:**

Nous avons examiné 34 articles décrivant 22 chaînes de température contrôlée ou d'autres expériences similaires dans quatre régions de l'Organisation mondiale de la Santé. Nous avons identifié une forte demande pour cette approche chez les fournisseurs de services; cependant, pour déclencher une demande identique chez les législateurs, il faut plus d'arguments axés sur les retombées économiques et l'amélioration de la couverture vaccinale, ainsi que des définitions de cas d'utilisation. De nombreux éléments probants ont confirmé que l'approche était sûre lorsqu'elle est intégrée dans des programmes de vaccination spéciaux. Assurer un niveau de formation et de supervision acceptable a permis aux fournisseurs de se conformer aux protocoles. Le gain de temps est le principal atout en termes d'efficacité, tandis que les données sur la réduction des coûts sont limitées. Stocker les vaccins au-delà de la chaîne du froid a rendu la couverture vaccinale plus équitable car les populations difficiles à atteindre ont ainsi pu y avoir accès. Rien n'a permis d'indiquer une diminution de l'efficacité des vaccins ou un taux d'effets indésirables plus élevé pour les vaccins fournis dans le cadre de cette approche.

**Conclusion:**

La synthèse des preuves a largement corroboré la théorie du changement. Apporter des arguments supplémentaires pour démontrer ses bienfaits en matière d'économie et de couverture vaccinale pourrait favoriser l'adhésion.

## Resumen

**Objetivo:**

Evaluar las pruebas que describen cómo el enfoque de la cadena de temperatura controlada para la vacunación podría suponer una mejora de la cobertura de inmunización equitativa en los países de ingresos bajos y medios.

**Métodos:**

Se creó un constructo de teoría del cambio a partir de la *Cadena de temperatura controlada: hoja de ruta estratégica para las vacunas prioritarias 2017-2020*, que contiene cuatro dominios: (i) adopción y demanda del enfoque; (ii) cumplimiento y uso seguro del enfoque; (iii) beneficios del enfoque en términos de eficacia del programa; y (iv) mejora de la cobertura de inmunización equitativa. Para verificar y mejorar la teoría del cambio, se aplicó un método de revisión realista para analizar las descripciones publicadas de la cadena de temperatura controlada o de experiencias muy relacionadas.

**Resultados:**

Se evaluaron 34 artículos, que describían 22 experiencias específicas de cadena de temperatura controlada o muy relacionadas en cuatro regiones de la Organización Mundial de la Salud. Se identificó una fuerte demanda de este enfoque entre los prestadores de servicios; sin embargo, para generar un nivel igual de demanda entre los responsables de formular políticas se requieren mayores pruebas sobre los beneficios económicos y de la cobertura de vacunación, así como definiciones de casos de uso. Las pruebas consistentes apoyan la seguridad del enfoque cuando se integra en programas especiales de vacunación. La formación y la supervisión factibles ayudaron a los prestadores a cumplir los protocolos. El ahorro de tiempo fue la principal prueba de los beneficios de eficiencia, mientras que los datos de ahorro de costes fueron mínimos. Se informó de una mayor cobertura equitativa cuando el almacenamiento de vacunas más allá de la cadena de frío permitió el acceso a poblaciones de difícil acceso. No hay pruebas que indiquen una menor eficacia de las vacunas ni un aumento de las tasas de efectos adversos de las vacunas suministradas mediante este enfoque.

**Conclusión:**

La síntesis de las pruebas apoyaron ampliamente la teoría inicial del cambio. Resolver las deficiencias de las pruebas sobre los beneficios económicos y de cobertura podría aumentar su adopción en el futuro.

## ملخص

**الغرض:**

تقييم الأدلة التي تصف كيف يمكن لأسلوب التطعيم بسلسلة درجات الحرارة الخاضعة للتحكم، أن يؤدي إلى تحسين تغطية التحصين المتكافئة في الدول ذات الدخل المنخفض والدخل المتوسط.

**الطريقة:**

لقد وضعنا نظرية لبناء التغيير من سلسلة درجات الحرارة الخاضعة للتحكم: خارطة طريق استراتيجية للقاحات ذات الأولوية 2017 إلى 2020، وتحتوي على أربعة قطاعات: (1) استيعاب الأسلوب والطلب عليه؛ و(2) الامتثال والاستخدام الآمن للأسلوب؛ و(3) مكاسب الكفاءة البرنامجية من هذا الأسلوب؛ و(4) تغطية التحصين المُحسّنة والمتكافئة. للتحقق من نظرية التغيير وتحسينها، قمنا بتطبيق طريقة مراجعة واقعية لتحليل التوصيفات المنشورة لسلسلة درجات الحرارة الخاضعة للتحكم، أو التجارب وثيقة الصلة.

**النتائج:**

قمنا بتقييم 34 مقالاً، تصف 22 سلسلة فريدة لدرجات الحرارة الخاضعة للتحكم، أو تجارب وثيقة الصلة عبر أربع مناطق تابعة لمنظمة الصحة العالمية. لقد حددنا طلبًا قويًا على مثل هذا الأسلوب بين جهات تقديم الخدمات؛ إلا أن توليد مستوى مكافئ من الطلب بين واضعي السياسات يتطلب أدلة أكبر على الفوائد الاقتصادية، ومكاسب تغطية التحصين، وتعريفات حالة الاستخدام. دعمت الأدلة المتسقة سلامة الأسلوب عند دمجها في برامج التحصين الخاصة. ساعد كل من التدريب العملي والإشراف جهات تقديم الخدمات في الامتثال للبروتوكولات. كان التوفير في الوقت هو الدليل الرئيسي على مكاسب الكفاءة، بينما كانت بيانات توفير التكاليف عند حدها الأدنى. تم الإبلاغ عن تغطية مُحسّنة ومتكافئة حينما أتاح تخزين اللقاح خارج سلسلة التبريد، الوصول إلى السكان الذين يصعب الوصول إليهم. لا يوجد دليل يشير إلى فعالية أقل للقاح، ولا زيادة معدلات الأحداث السلبية للقاحات التي يتم تقديمها بموجب هذا الأسلوب.

**الاستنتاج:**

دعمت الأدلة المجمعة بشكل واضح النظرية الأولية للتغيير. إن التعامل مع فجوات الأدلة على الفوائد الاقتصادية ومكاسب التغطية، قد يؤدي إلى زيادة الاستيعاب في المستقبل.

## 摘要

**目的:**

旨在评估有关疫苗受控温度链方法如何促使中低收入国家提高公平免疫接种率的证据。

**方法:**

我们根据*《可控温度链：2017-2020 年优先疫苗战略路线图》*创建了包含以下四个领域的变革理论：(i) 对该方法的理解和要求；(ii) 该方法的合规性和安全使用；(iii) 该方法带来的计划性效率提高；以及(iv) 提高公平免疫接种率。为了验证和改进该变革理论，我们采用现实主义综述方法来分析描述受控温度链或密切相关经验的已公布信息。

**结果:**

我们评估了 34 篇文章，了解了世界卫生组织四个地区的 22 种独特的受控温度链或密切相关经验。我们发现服务提供商强烈要求采用这种方法；但是，如要促使同样多的政策制定者认同此类需求，则需要有更多的证据来证明经济效益以及疫苗接种率提高和用例定义。证据一致性表明将该方法纳入特殊疫苗接种计划时能够确保安全性。切实可行的培训和监督支持提供商遵守协议。节省时间是表明效率提高的主要依据，但成本节约相关数据很少。据报道，如果非冷链存储疫苗能够供难以触及的人群使用，则可提高公平接种率。并无任何证据表明采用该方法提供的疫苗效果较差或不良事件发生率升高。

**结论:**

综合证据广泛支持初始变革理论。解决经济效益和接种率升高方面的证据差距可能会导致未来采用率提高。

## Резюме

**Цель:**

Оценить фактические данные, описывающие, как подход к вакцинации в рамках системы с регулируемой температурой может привести к обеспечению справедливого охвата иммунизацией в странах с низким и средним уровнем дохода.

**Методы:**

Авторы создали теорию преобразований на основе *системы с регулируемой температурой: стратегический план для приоритетных вакцин на 2017–2020 гг.* для четырех областей: (i) восприятие подхода и спрос на него; (ii) соблюдение и безопасное использование подхода; (iii) повышение эффективности программ благодаря этому подходу; (iv) улучшенный справедливый охват иммунизацией. Чтобы проверить и улучшить теорию преобразований, авторы применили метод реалистичного обзора для анализа опубликованных описаний системы с регулируемой температурой или подобного опыта.

**Результаты:**

Авторы проанализировали 34 статьи, описывающие 22 уникальные системы с регулируемой температурой или подобный опыт в четырех регионах Всемирной организации здравоохранения. Авторы выявили высокий спрос на такой подход среди поставщиков услуг, однако для создания равного уровня спроса со стороны лиц, ответственных за разработку политики, требуется больше фактических данных об экономической пользе и увеличении охвата вакцинацией, а также определении сценариев. Убедительные данные подтверждают безопасность этого подхода при его включении в специальные программы по вакцинации. Поставщикам услуг предоставлялось возможное обучение и контроль при соблюдении протоколов. Данные об экономии времени были основными данными повышения эффективности, в то время как данные об экономии расходов были минимальными. Сообщалось об улучшении справедливого охвата там, где хранение вакцин за пределами холодовой цепи обеспечивало доступ к труднодоступным группам населения. Нет данных, указывающих на более низкую эффективность вакцины или повышенную частоту нежелательных явлений для вакцин, вводимых в рамках данного подхода.

**Вывод:**

Объединенные данные в целом поддерживают первоначальную теорию преобразований. Устранение пробелов в фактических данных об экономической пользе и расширении охвата может увеличить вероятность использования подхода в будущем.

## Introduction

In many low- and middle-income countries, standard cold chain (2–8 °C) capacity for vaccine delivery is often restricted or unreliable,^1^ leading to vaccine stock-outs, increasing equipment costs and limiting the availability of vaccines in remote areas. During outreach campaigns, the use of carrier boxes and ice-packs to maintain a cold chain right to the point of vaccine administration increases time and cost, and risks vaccine damage by freezing through incorrectly placing vaccines in direct contact with ice-packs. A solution is the controlled temperature chain approach, a vaccine management protocol endorsed by the World Health Organization (WHO), which leverages the existing thermostability of certain vaccines to allow more flexibility in service delivery.^2^ By providing a safe and simple protocol for storage of selected vaccines at temperatures beyond the standard cold chain, the controlled temperature chain has the potential to substantially improve vaccination equity.^3^

For a vaccine to be used under a controlled temperature chain, manufacturers must demonstrate to regulators that exposure to temperatures ≥ 40 °C for a minimum three-days single planned excursion neither impedes vaccine safety nor effectiveness. Monitoring of exposures is mandated, requiring both vaccine vial monitors to measure cumulative heat exposure and peak temperature threshold indicators to measure instantaneous heat exposure. The controlled temperature chain approach is currently recommended only for special vaccine delivery strategies (e.g. births, school campaigns and outbreak response), with three vaccines currently WHO-prequalified for controlled temperature chain use. As of 2017, more than 4 million vaccines have been administered under this approach.^2^^,^^4^

The WHO *Controlled temperature chain: strategic roadmap for priority vaccines 2017–2020* provides a descriptive framework explaining how controlled temperature chain can lead to greater, and more equitable, immunization coverage for eligible vaccines.^2^ Here we use a realist review method to synthesize evidence from controlled temperature chain experiences to identify key priorities for future research. We also establish what may be needed to promote stakeholders’ interest for greater controlled temperature chain uptake in low- and middle-income countries.

## Methods

### Initial theory of change

We first obtained the descriptions of how the controlled temperature chain approach could lead to improved equitable immunization coverage from the *Controlled temperature chain: strategic roadmap for priority vaccines 2017–2020*, with descriptions supplemented from an associated commentary piece.^2^^,^^5^ Once obtained, we articulated these descriptions as a theory of change following a previously published context–mechanism–outcome construct.^6^^,^^7^ To ensure representativeness and accuracy of the articulated theory of change to source descriptions, we consulted the WHO Controlled Temperature Chain Working Group. Outcomes included in the theory of change were limited to implementation and excluded manufacturing considerations.

We commenced the evidence synthesis once a consensus on the initial theory of change had been reached.

### Evidence synthesis

#### Evidence search

We searched MEDLINE®, EMBASE®, CINAHL and Web of Science (all databases) using the key terms presented in Box 1, up to 7 April 2022. Targeted online searches, reference combing and contacting the WHO working group for additional evidence complemented the search. We applied no language restrictions.

Box 1 **Key terms used to identify studies on controlled temperature chain for vaccination**

- “controlled-temperature chain” or “controlled temperature chain” or “out-of-the-cold-chain” or “out-of-cold-chain” or “outside the cold chain” or “outside cold chain” or thermostable
- AND
- vaccine or vaccination or immuni$e or immuni$ation or HPV or OCV or Hepatitis B or Tetanus or Birth Dose or MenAfriVac or Meningitis

Studies using either controlled temperature chain or controlled temperature chain-relevant approaches (e.g. planned storage of non-controlled temperature chain-approved vaccines beyond the standard cold chain) were eligible for inclusion. We excluded perspectives from key stakeholders (e.g. commentary pieces, laboratory-based studies) or studies that were not based on an implementation experience. Economic modelling studies were considered eligible if costs were ascertained from a controlled temperature chain or controlled temperature chain-relevant implementation experience.

Two authors subjectively evaluated whether studies provided evidence on at least one aspect in the theory of change construct and the rigour of the evidence. To assess rigour in studies with sufficient methodological description, we used three quality assessment checklists; (i) Cochrane Effective Practice and Organization of Care for quantitative studies;^8^ (ii) Critical Appraisal Skills Programme for qualitative studies;^9^ and (iii) Consensus on Health Economic Criteria list for economic evaluations.^10^ Across all checklists, we classified quality of evidence on a three-tier (yes/no/unclear or high quality/low quality/unclear) scale.

#### Extraction and synthesis

Data extraction was thematic, guided by context–mechanism–outcome constructs within the initial theory of change, and supplemented by relevant categories from the WHO Supporting the Use of Research Evidence checklist.^11^ We did not apply any saturation threshold. Two authors initially extracted the text verbatim from source documents, and subsequently aggregated the text to identify key overlapping or contrasting concepts.

We employed narrative methods for the synthesis,^12^^,^^13^ allowing for aggregation of quantitative and qualitative findings. Two authors completed the synthesis, then it was cross-checked by other authors for consensus. Levels of supporting evidence for mechanisms and outcomes were subjectively determined, informed by quality and quantity of included studies. Evidence was deemed strong if supported by high-quality evidence and results repeated across multiple experiences. The synthesis was iterative and we revised the initial theory of change if supported by identified evidence.

The synthesis was adherent with reporting standards for realist syntheses^14^ and, where applicable, preferred reporting items for systematic reviews and meta-analyses.^15^ We registered the synthesis on OSF (https://osf.io/a3z6s).

## Results

**Fig. 1:**
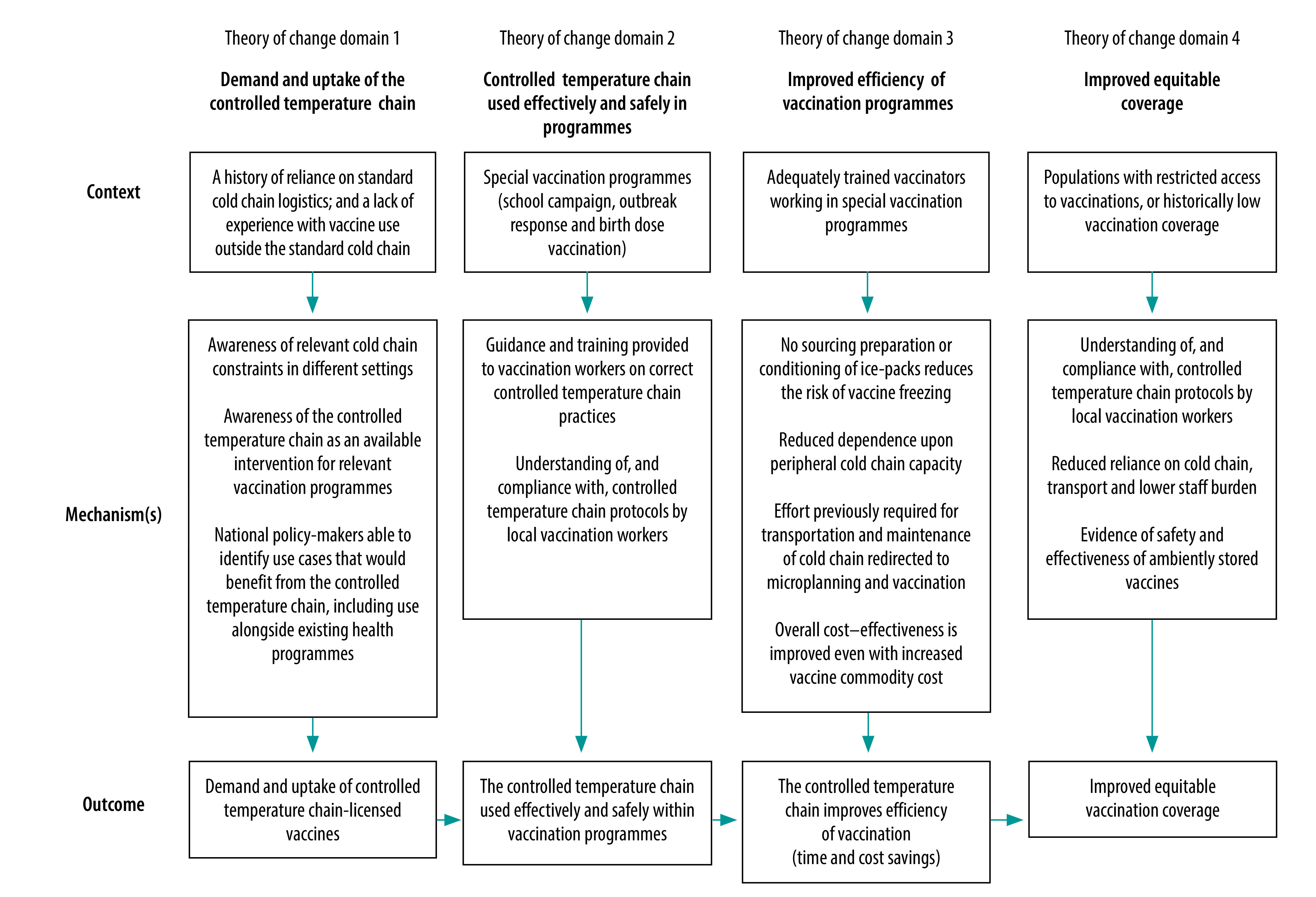
Context–mechanism–outcome construct for the theory of change of the Strategic Roadmap for Priority Vaccines

Note: We derived this construct from descriptions contained in the *Controlled temperature chain: strategic roadmap for priority vaccines 2017–2020*.^2^ Downward arrows indicate link of context–mechanism–outcome within each theory of change domain. Horizontal arrows link domains to each other within the overall theory of change.

The initial theory of change shows that due to the historic reliance on the standard cold chain, awareness of controlled temperature chain and relevant use cases are needed to drive demand and uptake (domain 1). Once safely and effectively implemented within special vaccination activities (domain 2), controlled temperature chain would enhance efficiency (domain 3) and equity of coverage (domain 4; Fig. 1). 

### Review of evidence

**Fig. 2:**
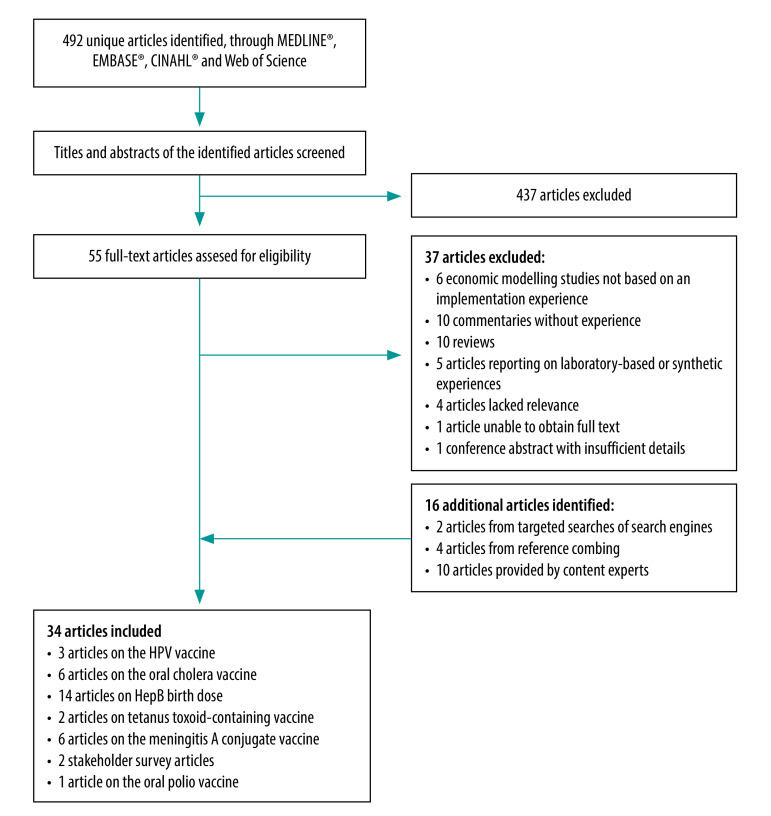
Study selection for the realist synthesis on the evidence of controlled temperature chain for vaccination in low- and middle-income countries

We identified 34 eligible articles, including 22 unique controlled temperature chain or relevant implementation descriptions (Fig. 2).^4^^,^^16^^–^^48^ Most frequently (14/34; 41%) articles covered hepatitis B (HepB) birth dose descriptions,^16^^–^^29^ but implementations were limited to the South-East Asia and Western Pacific Regions. Approximately one quarter of implementation experiences used a controlled temperature chain-licensed vaccine (6/22; 27%): four used meningitis A conjugate vaccine,^4^^,^^30^^–^^33^ one used human papillomavirus (HPV) vaccine^34^ and one used oral cholera vaccine.^35^ Although not designated a priority vaccine for controlled temperature chain,^2^ a single oral polio vaccine implementation study was eligible for synthesis.^36^ Almost half the experiences (10/22; 45%) were from the African Region,^4^^,^^30^^–^^34^^,^^36^^–^^40^^,^^42^^,^^44^^,^^45^ while no relevant experiences were identified in the Eastern Mediterranean or European Regions (Table 1).

HepB: hepatitis B; HPV: human papillomavirus.

Note: Sutanto et al. described a controlled temperature chain-relevant experience using both the HepB birth dose and the tetanus toxoid-containing vaccine.^16^

**Table 1:** Summary of studies included in synthesis of the contribution of controlled temperature chain for vaccine to the theory of change

Study	Location, WHO region	Vaccine	Brief summary	Contribution to theory of change domain			
				Demand and uptake	Safe and compliant use	Efficiency gains	Improved equitable vaccination coverage
Quiroga et al., 1998^47^	Bolivia (Plurinational State of), Region of the Americas	Tetanus toxoid-containing vaccine	Controlled temperature chain-relevant storage of compact pre-filled auto-disable devices used to facilitate vaccination of pregnant women at home during antenatal visits	No	Yes	Yes	No
Otto et al., 1999^17^	Indonesia, South-East Asia Region	HepB birth dose	Seroconversion comparison between controlled temperature chain-relevant stored compact pre-filled auto-disable devices and vaccines stored in the standard cold chain, measured after completion of infant vaccination series	No	Yes	Yes	Yes
Sutanto et al., 1999^16^	Indonesia, South-East Asia Region	HepB birth dose and tetanus toxoid-containing vaccine	Effectiveness of controlled temperature chain-relevant stored compact pre-filled auto-disable devices assessed, and health worker perceptions on controlled temperature chain-relevant storage gauged	Yes	Yes	Yes	No
Nelson et al., 2002^25^	Indonesia, South-East Asia Region	HepB birth dose	Perspective of midwives who used controlled temperature chain-relevant stored compact pre-filled auto-disable devices to deliver birth dose during home births in a rural setting	Yes	Yes	Yes	Yes
Levin et al., 2005^18^	Indonesia, South-East Asia Region	HepB birth dose	Economic evaluation of using controlled temperature chain-relevant stored compact pre-filled auto-disable devices to deliver the birth dose to home births in remote villages	Yes	Yes	Yes	Yes
PATH, 2005^27^	China, Western Pacific Region	HepB birth dose	Coverage, timeliness and effectiveness of controlled temperature chain-relevant stored vaccine compared with standard cold chain practices in a rural setting	Yes	Yes	Yes	Yes
Hipgrave et al., 2006^23^	Viet Nam, Western Pacific Region	HepB birth dose	Comparative immunogenicity after full vaccine series, with birth dose storage either controlled temperature chain-relevant or in standard cold chain	Yes	Yes	Yes	Yes
Huong et al., 2006^26^	Viet Nam, Western Pacific Region	HepB birth dose	Coverage, promptness and vaccine effectiveness of controlled temperature chain-relevant stored birth dose compared with the standard cold chain in a rural setting	Yes	Yes	Yes	Yes
Wang et al., 2007^21^	China, Western Pacific Region	HepB birth dose	Coverage, timeliness and effectiveness of controlled temperature chain-relevant stored vaccine compared with standard cold chain practices in a rural setting	Yes	Yes	Yes	Yes
Halm et al., 2010^36^	Mali, African Region	Oral polio vaccine	Vaccine wastage levels measured, and vaccinator preference assessed, for outreach vaccine delivery in a crossover intervention study comparing controlled temperature chain-relevant storage versus standard practice	Yes	Yes	Yes	Yes
Morgan et al., 2010^29^	Papua New Guinea, Western Pacific Region	HepB birth dose	Assessment of coverage, acceptability and feasibility of controlled temperature chain-relevant stored compact pre-filled auto-disable devices to enable village health volunteers to deliver birth doses during home births in a rural setting	Yes	Yes	No	Yes
Morgan et al., 2011^28^	Papua New Guinea, Western Pacific Region	HepB birth dose	Economic evaluation of controlled temperature chain-relevant stored compact pre-filled auto-disable devices delivered by village health volunteers to deliver birth doses during home births in a rural setting	No	No	Yes	Yes
Ciglenecki et al., 2013^42^	Guinea, African Region	Oral cholera vaccine	Controlled temperature chain-relevant transportation and storage of vaccine vials for outreach vaccination in a reactive campaign	Yes	Yes	Yes	Yes
Wigle et al., 2013^48^	Regions of those interviewed not stated	HPV vaccine	Key informant interviews used to ascertain greatest barriers to vaccine delivery, including potential barriers overcome by use of controlled temperature chain	Yes	No	No	No
Juan-Giner et al., 2014^37^	Chad, African Region	Tetanus toxoid-containing vaccine	Safety and effectiveness of controlled temperature chain-relevant stored vaccines compared with the standard cold chain in a non-inferiority trial	Yes	Yes	Yes	Yes
Luquero et al., 2014^44^	Guinea, African Region	Oral cholera vaccine	Case–control study estimating vaccine effectiveness of controlled temperature chain-relevant transported and stored vaccine in a reactive campaign	No	No	No	Yes
Lydon et al., 2014^45^	Chad, African Region	Meningitis A conjugate vaccine	Economic evaluation assessing incremental cost differences of using controlled temperature chain instead of the standard cold chain in a vaccine campaign	No	No	Yes	No
Porta et al., 2014^40^	South Sudan, African Region	Oral cholera vaccine	Description of a reactive vaccine campaign where controlled temperature chain-relevant storage and transportation of vials was used	Yes	Yes	Yes	Yes
Steffen et al., 2014^31^	Benin, African Region	Meningitis A conjugate vaccine	Comparison of adverse event rates from controlled temperature chain and cold chain stored vaccines, and average duration of controlled temperature chain assessed	No	Yes	Yes	Yes
Zipursky et al., 2014^4^	Benin, African Region	Meningitis A conjugate vaccine	Description of first prequalified controlled temperature chain vaccine experience, which includes a survey of vaccination staff for perceptions of the approach	Yes	Yes	Yes	Yes
Kolwaite et al., 2016^22^	Lao People's Democratic Republic, Western Pacific Region	HepB birth dose	Pilot study evaluating total coverage, timeliness and acceptability of controlled temperature chain-relevant storage compared with standard cold chain approach in two areas	Yes	Yes	Yes	Yes
Kouassi et al., 2016^33^	Côte d’Ivoire, African Region	Meningitis A conjugate vaccine	Knowledge of controlled temperature chain practices among vaccination staff and supervisors surveyed during a vaccine campaign	No	Yes	Yes	No
Kristensen et al., 2016^41^	Six countries from the African, American, South-East Asia and Western Pacific Regions	N/A	Stakeholders interviewed on their perspective towards thermostable vaccines, including the use of a controlled temperature chain	Yes	No	No	No
Ladner et al., 2016^43^	19 countries from African, American, European, South-East Asia and Western Pacific Regions	HPV vaccine	Questionnaire of key stakeholders in vaccine implementations to identify programme barriers, including those which could be overcome by a controlled temperature chain-licensed vaccine	Yes	No	No	No
Breakwell et al., 2017^20^	Solomon Islands, Western Pacific Region	HepB birth dose	Controlled temperature chain-relevant storage piloted in remote health facilities. Health workers surveyed on perceived acceptability, feasibility and barriers of this approach	Yes	Yes	Yes	Yes
Landoh et al., 2017^32^	Togo, African Region	Meningitis A conjugate vaccine	Comparative coverage of the vaccine in controlled temperature chain and standard cold chain assigned areas evaluated using a cluster randomized survey	Yes	Yes	No	Yes
Li et al., 2017^24^	Kiribati, Western Pacific Region	HepB birth dose	Controlled temperature chain-relevant storage of birth dose encouraged to help increase coverage among home births	Yes	Yes	No	Yes
Mvundura et al., 2017^30^	Togo, African Region	Meningitis A conjugate vaccine	Economic evaluation of incremental supply chain costs for the vaccine when used in controlled temperature chain compared with the standard cold chain during a campaign	Yes	No	Yes	No
Petit et al., 2017^19^	African and Western Pacific Regions	HepB birth dose	Vaccination stakeholders questioned about interest, perceived benefits and willingness-to-pay for a controlled temperature chain-licensed vaccine	Yes	No	No	No
Grandesso et al., 2018^38^	Malawi, African Region	Oral cholera vaccine	Controlled temperature chain-relevant storage of vaccine vials used to facilitate self-administration of second dose in a remote population	Yes	Yes	Yes	Yes
Heyerdahl et al., 2018^39^	Malawi, African Region	Oral cholera vaccine	In-depth interviews and focus groups used to investigate acceptability of controlled temperature chain-relevant storage to facilitate self-administration of second dose in a remote population	Yes	Yes	No	No
WHO, 2018^34^	Uganda, African Region	HPV vaccine	Pilot study comparing worker perceptions, coverage, vaccine wastage and efficiency of vaccine under controlled temperature chain versus standard cold chain for a school-based campaign in a rural setting	Yes	Yes	Yes	Yes
Khan et al., 2019^35^	Bangladesh, South-East Asia Region	Oral cholera vaccine	Evaluation of coverage, safety and acceptability of controlled temperature chain-licensed vaccines when used to facilitate self-administration of second dose at home	Yes	Yes	Yes	Yes
Mvundura et al., 2021^46^	61 countries, 75% from African Region	N/A	Qualitative study of vaccination stakeholders as part of the Vaccine Innovation Prioritisation Strategy, including questions on perceived benefits and use of controlled temperature chain vaccines	Yes	No	No	No

HepB: Hepatitis B; HPV: Human papillomavirus; N/A: not applicable (stakeholder perspective on the controlled temperature chain evaluated and was not limited to specific vaccines). WHO: World Health Organization.

Note: Relevant approaches refers to vaccine use beyond the standard cold chain with a non-controlled temperature chain-approved vaccine. More details are available in the data repository.^49^

Six articles had quality assessment domains at a high risk of bias or with an unmet criterion.^20^^,^^22^^,^^28^^,^^33^^,^^38^^,^^41^ Most included studies had one or more domains where evidence quality was unclear. Further details are available in the data repository.^49^

#### Theory of change domain 1

##### Demand and uptake

The key context identified for the demand and uptake of the approach was appreciation of disease burden and a need to overcome cold chain limitations to vaccinate hard-to-reach populations. Appreciation of these standard cold chain limitations were reported in many implementation experiences,^4^^,^^20^^–^^23^^,^^26^^,^^32^^,^^37^^,^^42^ but by only 24% (6/25) of national stakeholders in a survey assessing interest for controlled temperature chain for prequalified HepB vaccines.^35^ We anticipated vaccine damage due to cold chain failures (freezing or heat exposure) to be a contextual driver of controlled temperature chain demand. While in two surveys, national and global stakeholders saw utility of such approach to avert these problems,^41^^,^^46^ vaccine damage as a driver of demand was only cited in four unique implementations.^16^^,^^17^^,^^21^^,^^34^^,^^36^

Some evidence supported the awareness of controlled temperature chain as a mechanism to drive demand and uptake among policy-makers. Three surveys indicated that 72% (18/25) to 75% (21/28) of national and global policy-makers showed a demand for controlled temperature chain use.^19^^,^^41^^,^^46^ Awareness raised through policy adaptations, including WHO endorsement for controlled temperature chain-relevant storage of the HepB birth dose, predicated uptake in two implementations.^20^^,^^22^ Awareness via endorsement from implementation partners was noted to influence uptake by health ministries in oral cholera vaccine and tetanus toxoid-containing vaccine experiences.^37^^,^^40^

There was limited evidence on identification of credible and beneficial use cases for controlled temperature chain vaccines, a mechanism required to drive sustained demand by policy-makers. We identified a clearly defined use case for meningitis A conjugate vaccine across four unique experiences: campaigns in remote sub-Saharan Africa with limited or no access to a cold chain.^4^^,^^30^^–^^33^ For other vaccines the evidence was emerging or yet to be determined. For example, controlled temperature chain facilitated self-administration of a second oral cholera dose or vaccination to be completed alongside more traditional cholera control strategies.^35^^,^^38^^,^^40^^,^^42^ We could not identify agreed controlled temperature chain use cases for the HepB vaccine despite 10 controlled temperature chain-relevant implementations.^16^^–^^18^^,^^20^^–^^24^^,^^26^^–^^29^ Even with a controlled temperature chain-licensed vaccine and awareness of relevant coverage barriers in HPV vaccination efforts,^43^^,^^48^ we only identified a single implementation.^34^

For vaccines with no defined use case, 20% (36/183) of respondents across two surveys saw the controlled temperature chain as a fall back mechanism for transient cold chain breaks.^19^^,^^41^ Sustained demand, via controlled temperature chain-licensure, varied across controlled temperature chain-relevant experiences; noted in three oral cholera vaccine experiences but only in a single HepB vaccine experience.^22^^,^^38^^,^^40^^,^^42^ In at least four experiences, controlled temperature chain-relevant vaccine storage enabled integration of vaccination into existing health programmes.^24^^,^^28^^,^^29^^,^^40^^,^^42^ However, fears of higher vaccine procurement costs, training feasibility and use of controlled temperature chain leading to poor cold chain practices dampened stakeholders’ demand for the controlled temperature chain.^19^^,^^41^^,^^46^

#### Theory of change domain 2

##### Safe and compliant use

**Fig. 3:**
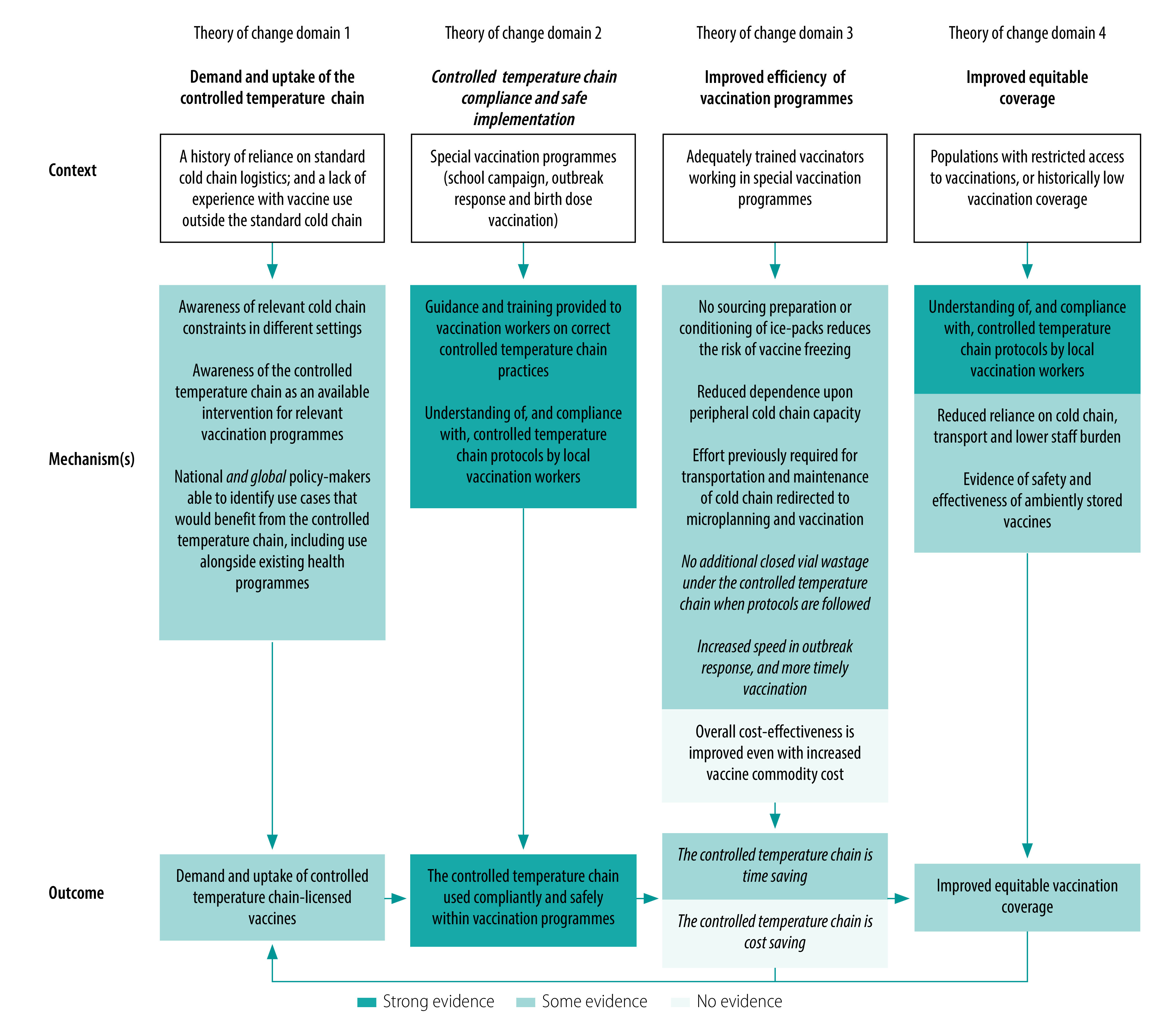
Revised context–mechanism–outcome construct for the theory of change of controlled temperature chain for vaccination in low- and middle-income countries

Note: Text in italics is revision of the initial theory of change in Fig. 1. Downward arrows indicate link of context–mechanism–outcome within each theory of change domain. Horizontal arrows link domains to each other within the overall theory of change.

Based on the evidence synthesis, we reframed this domain to focus on evidence of safe and compliant controlled temperature chain implementation (Fig. 3).

Within the context of special vaccination programmes, presence of temperature exposure monitoring technologies mediated safe and compliant vaccine use, including when used by health volunteers or by community members self-administering vaccines.^28^^,^^29^^,^^35^^,^^38^^,^^39^ Ten studies relied on vaccine vial monitors measuring cumulative heat exposures as the sole indicator of vaccine integrity.^18^^–^^22^^,^^36^^,^^38^^–^^40^^,^^42^ Five experiences used peak temperature threshold indicators to measure instantaneous heat exposures,^4^^,^^16^^,^^17^^,^^31^^,^^34^ while digital thermometers or comparison to ambient temperatures were also used in four experiences.^22^^,^^23^^,^^36^^,^^47^ In addition, use of monitoring forms to track vaccine use and exposures over an implementation was reported in four experiences.^4^^,^^33^^,^^34^^,^^36^

Strong and consistent evidence demonstrated that training of vaccinators in safe and compliant use of controlled temperature chain was feasible and nononerous. Training was often less than a day in duration or integrated among other programmatic activities.^16^^,^^22^^,^^29^^,^^33^^,^^34^^,^^47^ Training encompassed vaccine vial monitor interpretation, controlled temperature chain protocol awareness and use of peak temperature threshold indicators. While methodological rigour in evaluation of training success varied across experiences, and some protocols deviated from currently accepted controlled temperature chain standards,^16^^,^^17^^,^^36^^,^^42^ good compliance was consistently reported across all professional levels. Supervision, facilitated by implementation partners^4^^,^^34^^,^^42^ or project managers,^28^^,^^29^ was feasible and important for maintenance of correct practices and maximizing benefits of the controlled temperature approach. Examples included provision of real-time feedback to maximize efficiency during a meningitis A conjugate vaccine implementation in Benin,^4^ and identification and rectification of unsafe vaccine disposal by health volunteers in Papua New Guinea.^29^ Furthermore, supervision and training were seen as potential mechanisms to catalyse use of the controlled temperature approach where permitted in Kiribati.^24^ Further, no evidence suggested that the approach adversely affected other cold chain practices; however, we could only discern this interpretation from two implementations.^4^^,^^34^

#### Theory of change domain 3

##### Programme efficiency

The driver of improving programme efficiency and timeliness was existing inefficiencies in maintaining a standard cold chain for special vaccination programmes. Challenges and costs associated with providing cold chains for timely service delivery were described in studies on meningitis A conjugate vaccine and oral cholera vaccine experiences.^4^^,^^30^^,^^31^^,^^40^^,^^42^ Controlled temperature approaches were considered a more efficient alternative, linking to a demand for uptake. This feedback loop – efficiency of controlled temperature chain to overcome cold chain challenges leading to increased demand – is now reflected within the revised theory of change (Fig. 3).

This feedback loop was also supported by some evidence of a vaccinator preference (implying a demand) for controlled temperature chain or controlled temperature chain-relevant approaches over the standard cold chain. This preference was driven by efficiency related mechanisms, namely reported time-savings from no longer having to prepare, carry and replenish ice-packs during outreach vaccination efforts.^4^^,^^34^^,^^36^ A controlled temperature chain was generally evaluated as not adding any additional complexity to service provision, except in one experience where the increased frequency of vaccine replenishment was reported to increase workload.^20^

Little evidence existed in quantifying reduced costs of vaccination under the approach. Two implementations using HepB birth doses showed controlled temperature chain-relevant use of compact pre-filled auto-disable devices was more efficient than standard cold chain approaches, but savings were driven by devices-mediated task-shifting and waste reduction and could not be directly attributed to the controlled temperature chain.^18^^,^^28^ As prequalified controlled temperature chain vaccine experiences were limited, we could not assess trade-offs between higher vaccine costs and reduced cold chain costs, a concern raised in interviews with key stakeholders.^19^^,^^41^ Two studies estimated credible cost savings within meningitis A conjugate vaccine experiences, ignoring vaccine prices, under an assumption that a proportion of cold chain costs were avoided when using the controlled temperature chain.^30^^,^^45^ However, a direct comparison in one experience showed no incremental cost differences between the two approaches.^30^

Evidence supported two new mechanisms by which controlled temperature chain enhanced vaccination efficiency and timeliness. First, the approach enabled more rapid delivery of vaccination to target populations.^18^^,^^28^^,^^29^^,^^38^^,^^40^^,^^42^ Second, the approach was not associated with any additional vaccine wastage when compared with cold chain,^30^^,^^36^ and where any measurable wastage of vaccines stored beyond the cold chain occurred, it resulted from stock management and microplanning failures.^20^^,^^34^

We revised the key outcome for this domain in two elements after evidence synthesis (Fig. 3). Time-savings were reported, but not quantified, while no implementation study quantified cost savings. Credible but theoretical cost savings attributable to controlled temperature chain were extrapolated from modelling;^30^^,^^45^ and where cost savings were reported, causality could not be disentangled from the use of compact pre-filled auto-disable devices.^18^^,^^28^

#### Theory of change domain 4

##### Equitable vaccination coverage

The ability of the controlled temperature chain approach to improve equitable vaccination coverage was supported by promising evidence; however, experiences designed to quantify coverage gains were restricted to HepB birth vaccination.^20^^–^^22^^,^^26^^,^^27^ Coverage benefits varied by implementation setting: in Lao People's Democratic Republic, coverage gains were greatest for births in health facilities;^22^ whereas in rural China, the approach was most beneficial for timely coverage of home births.^21^ In some studies, observed coverage gains due to the approach were cited as motivation for uptake in other experiences,^20^^–^^22^ forming a feedback loop between equitable coverage gains and controlled temperature chain uptake. This feedback loop is now reflected in the revised theory of change (Fig. 3). Across controlled temperature chain and controlled temperature chain-relevant HPV, oral cholera and meningitis A conjugate vaccine experiences, reported high levels of coverage were unlikely to be achieved unless vaccines were stored beyond the cold chain.^4^^,^^32^^,^^35^^,^^40^

We found no evidence of the approach increasing adverse event rates or reducing vaccine effectiveness.^17^^,^^19^^–^^21^^,^^23^^,^^31^^,^^36^^–^^38^^,^^44^ Further, researchers for two studies in Viet Nam hypothesized that controlled temperature chain-relevant storage of the HepB birth dose have enhanced immunogenicity due to prevention of freezing.^23^^,^^26^

## Discussion

We identified a credible evidence base that broadly supported the initial theory of change; however, synthesis identified some key refinements. Evidence supported feasibility of safe controlled temperature chain integration into vaccination programmes, with robust evidence showing complicit and safe use by vaccinators. However, clearly defined use cases for most controlled temperature chain-eligible vaccines were lacking. Future research priorities to promote uptake of controlled temperature chain approach should include economic evaluations and studies to quantify equitable coverage gains.

Meningitis A conjugate vaccine delivery under the controlled temperature chain was only implemented in the African Region, probably due to the fact that the vaccine is designed for use in the sub-Saharan meningitis belt.^50^ Implementations of controlled temperature chain-relevant approach for the HepB birth dose were limited to the Western Pacific and South-East Asia regions. One reason may be the prominence of vertical transmission of HepB in these regions as compared with the African Region,^51^ and a generally low adoption and scale-up of HepB birth dose in the African Region.^52^ Another reason may be national and regional frameworks endorsing controlled temperature chain-relevant birth dose use in Western Pacific and South-East Asia regions,^24^^,^^53^ raising awareness and promoting uptake. Policy-makers should remain open to similar adaptations for other eligible vaccines, especially given available evidence on safe and compliant use.

Our synthesis identified robust evidence supporting the safe integration and compliant use of controlled temperature chain by vaccinators. Despite stakeholder concerns about costs or feasibility of training, evidence indicated the training can be integrated into other routine programme trainings or is of low burden when completed in a stand-alone fashion. Observed compliance with protocols was high across a wide range of health-education levels, including community members and volunteers. In addition, despite stakeholder concern, no evidence suggested practice of the approach led to miscreant cold chain practices. Evidence on safe and compliant use should encourage policy-makers to explore use cases where controlled temperature chain is the only option, such as storage of the HepB birth dose in remote locations or self-administered oral cholera vaccine. These use cases could both generate a sustained demand and facilitate more equitable, or timelier, vaccination coverage.

One key refinement made to the initial theory of change, shown as a feedback loop, was that efficiency of controlled temperature was driving demand. Decision-makers thought the approach overcame the problems of maintaining the cold chain in challenging circumstances and vaccinators preferred the decreased workload compared with standard cold chain. While few studies described averted freeze damage due to controlled temperature chain during implementations, we note that freeze damage is a common occurrence in many cold chains and poorly recognized by service providers,^54^^–^^56^ and performance gains on this aspect may have gone unreported in experiences. However, policy-makers hesitated regarding the potentially higher vaccine prices for a prequalified controlled temperature chain vaccine. We found no real-world implementation evidence to counter this hesitancy; rather, any evidence of cost–effectiveness (derived through gains in vaccine delivery efficiency) are currently derived from extrapolations or theoretical modelling.^30^^,^^45^^,^^57^^,^^58^ Given noted hesitations, future research should cover this area to help generate demand for the controlled temperature chain.

Another key refinement made to the theory of change was identification of a feedback loop between demand and equitable coverage gains. We observed that coverage gains attributable to controlled temperature chain-relevant storage were a driver of uptake in some experiences, and may provide a sense of confidence that the approach will be beneficial. However, more studies which quantify the direct coverage gains attributable to a controlled temperature chain approach are required. Demand for controlled temperature chain may increase if a causal link can be established between coverage gains and the approach, and not from an enhanced effort as occurs in a pilot study context.

Our synthesis has some key limitations. First, we limited our scope to uptake and implementation experiences and did not seek to include experiences of manufacturers or developers. While important and a potential avenue for future research, manufacturers’ willingness to develop or relicense vaccines for the controlled temperature chain will likely depend upon demand, a key focus of this synthesis. Second, we cannot exclude publication bias from synthesis findings. We did not identify any failed experiences. If these failures occurred, the possibility exists they did not get reported. Finally, realist methods are inherently subjective, and findings could be influenced by researcher perspectives. In an attempt to counteract this, results were frequently communicated to research commissioners and other experts for cross-checking.

Synthesis of evidence from controlled temperature chain approaches broadly supported the existing theory of change. Credible evidence demonstrated the overall feasibility of controlled temperature chain to improve equitable vaccination coverage in low- and middle-income countries, as well as supporting that integration of the approach into vaccination programmes is safe. Future research should conduct use case studies for eligible vaccines and quantify the economic and attributable coverage benefits of the approach in a range of health systems.

## References

[1] Next-generation immunization supply chains are needed to improve health outcomes. Seattle: PATH; 2015. Available from: https://path.azureedge.net/media/documents/APP_isc_key_messages_rptv2.pdf [cited 2021 May 6].

[2] Controlled temperature chain: strategic roadmap for priority vaccines 2017–2020. Geneva: World Health Organization; 2017. Available from: https://apps.who.int/iris/handle/10665/272994 [cited 2020 Jun 18].

[3] Patel MK, Kahn A-L. Game changing: hepatitis B vaccine in a controlled temperature chain. Lancet Glob Health. 2018 Jun;6(6):e596–7. 10.1016/S2214-109X(18)30233-X 29773113

[4] Zipursky S, Djingarey MH, Lodjo J-C, Olodo L, Tiendrebeogo S, Ronveaux O. Benefits of using vaccines out of the cold chain: delivering meningitis A vaccine in a controlled temperature chain during the mass immunization campaign in Benin. Vaccine. 2014 Mar 14;32(13):1431–5. 10.1016/j.vaccine.2014.01.038 24559895 PMC5355207

[5] Kahn AL, Kristensen D, Rao R. Extending supply chains and improving immunization coverage and equity through controlled temperature chain use of vaccines. Vaccine. 2017 Apr 19;35(17):2214–6. 10.1016/j.vaccine.2016.10.091 28364934

[6] Pawson R, Greenhalgh T, Harvey G, Walshe K. Realist synthesis: an introduction. Manchester: ESRC Research Methods Programme, University of Manchester; 2004.

[7] Pawson R, Greenhalgh T, Harvey G, Walshe K. Realist review–a new method of systematic review designed for complex policy interventions. J Health Serv Res Policy. 2005 Jul;10 Suppl 1:21–34. 10.1258/1355819054308530 16053581

[8] Suggested risk of bias criteria for EPOC reviews. CITY: Cochrane Effective Practice and Organisation of Care (EPOC); YEAR. Available from: https://epoc.cochrane.org/sites/epoc.cochrane.org/files/public/uploads/Resources-for-authors2017/suggested_risk_of_bias_criteria_for_epoc_reviews.pdf [cited 2020 Aug 15].

[9] CASP Checklist: 10 questions to help you make sense of a qualitative research. Oxford: Critical Appraisal Skills Programme; 2018. Available from: https://casp-uk.net/wp-content/uploads/2018/01/CASP-Qualitative-Checklist-2018.pdf [cited 2020 Aug 15].

[10] Evers S, Goossens M, de Vet H, van Tulder M, Ament A. Criteria list for assessment of methodological quality of economic evaluations: Consensus on Health Economic Criteria. Int J Technol Assess Health Care. 2005 Spring;21(2):240–5. 10.1017/S0266462305050324 15921065

[11] SURE Guides for Preparing and Using Evidence-Based Policy Briefs. CITY: SURE Collaboration; 2011. Available from: https://epoc.cochrane.org/sites/epoc.cochrane.org/files/public/uploads/SURE-Guides-v2.1/Collectedfiles/sure_guides.html [cited 2020 Oct 6].

[12] Popay J, Roberts H, Sowden A, Petticrew M, Arai L, Rodgers M, et al. Guidance on the conduct of narrative synthesis in systematic reviews. A product from the ESRC methods programme Version. Lancaster: Lancaster University; 2006. 10.13140/2.1.1018.4643

[13] Snilstveit B, Oliver S, Vojtkova M. Narrative approaches to systematic review and synthesis of evidence for international development policy and practice. J Dev Effect. 2012;4(3):409–29. 10.1080/19439342.2012.710641

[14] Wong G, Greenhalgh T, Westhorp G, Buckingham J, Pawson R. RAMESES publication standards: realist syntheses. BMC Med. 2013 Jan 29;11(1):21. 10.1186/1741-7015-11-21 23360677 PMC3558331

[15] Page MJ, McKenzie JE, Bossuyt PM, Boutron I, Hoffmann TC, Mulrow CD, et al. Updating guidance for reporting systematic reviews: development of the PRISMA 2020 statement. J Clin Epidemiol. 2021 Jun;134:103–12. 10.1016/j.jclinepi.2021.02.003 33577987

[16] Sutanto A, Suarnawa IM, Nelson CM, Stewart T, Soewarso TI. Home delivery of heat-stable vaccines in Indonesia: outreach immunization with a prefilled, single-use injection device. Bull World Health Organ. 1999;77(2):119–26. 10083709 PMC2557593

[17] Otto BF, Suarnawa IM, Stewart T, Nelson C, Ruff TA, Widjaya A, et al. At-birth immunisation against hepatitis B using a novel pre-filled immunisation device stored outside the cold chain. Vaccine. 1999 Oct 14;18(5-6):498–502. 10.1016/S0264-410X(99)00242-X 10519939

[18] Levin CE, Nelson CM, Widjaya A, Moniaga V, Anwar C. The costs of home delivery of a birth dose of hepatitis B vaccine in a prefilled syringe in Indonesia. Bull World Health Organ. 2005 Jun;83(6):456–61. 15976897 PMC2626261

[19] Petit D, Tevi-Benissan C, Woodring J, Hennessey K, Kahn A-L. Countries’ interest in a hepatitis B vaccine licensed for the controlled temperature chain; survey results from African and Western Pacific regions. Vaccine. 2017 Dec 14;35(49) 49 Pt B:6866–71. 10.1016/j.vaccine.2017.10.025 29132994 PMC5722051

[20] Breakwell L, Anga J, Dadari I, Sadr-Azodi N, Ogaoga D, Patel M. Evaluation of storing hepatitis B vaccine outside the cold chain in the Solomon Islands: Identifying opportunities and barriers to implementation. Vaccine. 2017 May 15;35(21):2770–4. 10.1016/j.vaccine.2017.04.011 28431814 PMC5893327

[21] Wang L, Li J, Chen H, Li F, Armstrong GL, Nelson C, et al. Hepatitis B vaccination of newborn infants in rural China: evaluation of a village-based, out-of-cold-chain delivery strategy. Bull World Health Organ. 2007 Sep;85(9):688–94. 10.2471/BLT.06.037002 18026625 PMC2636406

[22] Kolwaite AR, Xeuatvongsa A, Ramirez-Gonzalez A, Wannemuehler K, Vongxay V, Vilayvone V, et al. Hepatitis B vaccine stored outside the cold chain setting: a pilot study in rural Lao PDR. Vaccine. 2016 Jun 14;34(28):3324–30. 10.1016/j.vaccine.2016.03.080 27040399 PMC8735871

[23] Hipgrave DB, Tran TN, Huong VM, Dat DT, Nga NT, Long HT, et al. Immunogenicity of a locally produced hepatitis B vaccine with the birth dose stored outside the cold chain in rural Vietnam. Am J Trop Med Hyg. 2006 Feb;74(2):255–60. 10.4269/ajtmh.2006.74.255 16474080

[24] Li X, Heffelfinger J, Wiesen E, Diorditsa S, Valiakolleri J, Nikuata AB, et al. Improving hepatitis B birth dose coverage through village health volunteer training and pregnant women education. Vaccine. 2017 Aug 3;35(34):4396–401. 10.1016/j.vaccine.2017.06.056 28688784

[25] Nelson C, Widjaya A, Wittet S. Using Uniject™ to increase the safety and effectiveness of hepatitis B immunization. Occasional paper no. 6. Seattle: PATH; 2002.

[26] Huong VM, Hipgrave D, Hills S, Nelson C, Hien DS, Cuong N. Out-of-cold-chain delivery of the Hepatitis B birth dose in four districts of Vietnam. Seattle: PATH; 2006. Available from: https://path.azureedge.net/media/documents/TS_hepb_coldchain_vietnam.pdf [cited 2020 Oct 7].

[27] Study summary: evaluation of out-of-the-cold-chain approaches for improving on-time delivery of the hepatitis B birth dose in rural areas of China. Seattle: PATH; 2005. Available from: https://path.azureedge.net/media/documents/TS_hepB_coldchain_china.pdf [cited 2020 Oct 7].

[28] Morgan C, Rahadi A. Cost and outcomes analysis - improving immunisation and newborn survival at the aid post level in Papua New Guinea. Melbourne: Burnet Institute; 2011.

[29] Morgan C, Bisibisera L, Bauze A. Improving immunisation and newborn survival at the aid post level in Papua New Guinea. Melbourne: Burnet Institute; 2010.

[30] Mvundura M, Lydon P, Gueye A, Diaw IK, Landoh DE, Toi B, et al. An economic evaluation of the controlled temperature chain approach for vaccine logistics: evidence from a study conducted during a meningitis A vaccine campaign in Togo. Pan Afr Med J. 2017 Jun 23;27 Suppl 3:27. 10.11604/pamj.supp.2017.27.3.12087 29296162 PMC5745944

[31] Steffen C, Tokplonou E, Jaillard P, Dia R, Alladji MNDB, Gessner B. A field based evaluation of adverse events following MenAfriVac® vaccine delivered in a controlled temperature chain (CTC) approach in Benin. Pan Afr Med J. 2014 Aug 28;18:344. 25574320 10.11604/pamj.2014.18.344.3975 PMC4282804

[32] Landoh DE, Kahn AL, Lacle A, Adjeoda K, Saka B, Yaya I, et al. L’utilisation de l’approche CTC: quel impact sur la couverture vaccinale lors de la campagne préventive de vaccination contre la méningite A avec le MenAfriVac au Togo en 2014? Pan Afr Med J. 2017 May 12;27:38. [French]. 10.11604/pamj.2017.27.38.11873 28761614 PMC5516651

[33] Kouassi DP, Aka LBN, Bénié BVJ, Coulibaly SR, Tagodé DB, Coulibaly D, et al. Practice of controlled temperature chain (CTC) technique during a mass vaccination campaign in Côte d’Ivoire. World J Vaccines. 2016;6(1):16–22. 10.4236/wjv.2016.61003

[34] Using the HPV vaccine in a controlled temperature chain (CTC) in Uganda - a pilot project. Geneva: World Health Organization; 2018.

[35] Khan AI, Islam MS, Islam MT, Ahmed A, Chowdhury MI, Chowdhury F, et al. Oral cholera vaccination strategy: self-administration of the second dose in urban Dhaka, Bangladesh. Vaccine. 2019 Feb 4;37(6):827–32. 10.1016/j.vaccine.2018.12.048 30639459

[36] Halm A, Yalcouyé I, Kamissoko M, Keïta T, Modjirom N, Zipursky S, et al. Using oral polio vaccine beyond the cold chain: a feasibility study conducted during the national immunization campaign in Mali. Vaccine. 2010 Apr 26;28(19):3467–72. 10.1016/j.vaccine.2010.02.066 20197147

[37] Juan-Giner A, Domicent C, Langendorf C, Roper MH, Baoundoh P, Fermon F, et al. A cluster randomized non-inferiority field trial on the immunogenicity and safety of tetanus toxoid vaccine kept in controlled temperature chain compared to cold chain. Vaccine. 2014 Oct 29;32(47):6220–6. 10.1016/j.vaccine.2014.09.027 25261378

[38] Grandesso F, Rafael F, Chipeta S, Alley I, Saussier C, Nogareda F, et al. Oral cholera vaccination in hard-to-reach communities, Lake Chilwa, Malawi. Bull World Health Organ. 2018 Dec 1;96(12):817–25. 10.2471/BLT.17.206417 30505029 PMC6249704

[39] Heyerdahl LW, Ngwira B, Demolis R, Nyirenda G, Mwesawina M, Rafael F, et al. Innovative vaccine delivery strategies in response to a cholera outbreak in the challenging context of Lake Chilwa. A rapid qualitative assessment. Vaccine. 2018 Oct 22;36(44):6491–6. 10.1016/j.vaccine.2017.10.108 29126808 PMC6189868

[40] Porta MI, Lenglet A, de Weerdt S, Crestani R, Sinke R, Frawley MJ, et al. Feasibility of a preventive mass vaccination campaign with two doses of oral cholera vaccine during a humanitarian emergency in South Sudan. Trans R Soc Trop Med Hyg. 2014 Dec;108(12):810–15. 10.1093/trstmh/tru153 25311798

[41] Kristensen DD, Lorenson T, Bartholomew K, Villadiego S. Can thermostable vaccines help address cold-chain challenges? Results from stakeholder interviews in six low- and middle-income countries. Vaccine. 2016 Feb 10;34(7):899–904. 10.1016/j.vaccine.2016.01.001 26778422 PMC4744085

[42] Ciglenecki I, Sakoba K, Luquero FJ, Heile M, Itama C, Mengel M, et al. Feasibility of mass vaccination campaign with oral cholera vaccines in response to an outbreak in Guinea. PLoS Med. 2013;10(9):e1001512. 10.1371/journal.pmed.1001512 24058301 PMC3769208

[43] Ladner J, Besson M-H, Audureau E, Rodrigues M, Saba J. Experiences and lessons learned from 29 HPV vaccination programs implemented in 19 low and middle-income countries, 2009–2014. BMC Health Serv Res. 2016 Oct 13;16(1):575. 10.1186/s12913-016-1824-5 27737666 PMC5062879

[44] Luquero FJ, Grout L, Ciglenecki I, Sakoba K, Traore B, Heile M, et al. Use of Vibrio cholerae vaccine in an outbreak in Guinea. N Engl J Med. 2014 May 29;370(22):2111–20. 10.1056/NEJMoa1312680 24869721

[45] Lydon P, Zipursky S, Tevi-Benissan C, Djingarey MH, Gbedonou P, Youssouf BO, et al. Economic benefits of keeping vaccines at ambient temperature during mass vaccination: the case of meningitis A vaccine in Chad. Bull World Health Organ. 2014 Feb 1;92(2):86–92. 10.2471/BLT.13.123471 24623901 PMC3949534

[46] Mvundura M, Frivold C, Janik Osborne A, Soni P, Robertson J, Kumar S, et al. Vaccine innovation prioritisation strategy: findings from three country-stakeholder consultations on vaccine product innovations. Vaccine. 2021 Dec 3;39(49):7195–207. 10.1016/j.vaccine.2021.08.024 34412922 PMC8657797

[47] Quiroga R, Halkyer P, Gil F, Nelson C, Kristensen D. A prefilled injection device for outreach tetanus immunization by Bolivian traditional birth attendants. Rev Panam Salud Publica. 1998 Jul;4(1):20–5. 10.1590/S1020-49891998000700004 9734224

[48] Wigle J, Coast E, Watson-Jones D. Human papillomavirus (HPV) vaccine implementation in low and middle-income countries (LMICs): health system experiences and prospects. Vaccine. 2013 Aug 20;31(37):3811–17. 10.1016/j.vaccine.2013.06.016 23777956 PMC3763375

[49] Seaman CP, Kahn AL, Kristensen D, Steinglass R, Spasenoska D, Scott N, et al. Supplemental information for: Use of the controlled-temperature chain for vaccination in low and middle-income countries– a realist evidence synthesis of uptake and impact determinants [data repository]. Meyrin: Zenodo; 2022. 10.5281/zenodo.6583035 10.5281/zenodo.6583035 PMC9306389 35923285

[50] Berlier M, Barry R, Shadid J, Sirica C, Brunier A, Hasan H, et al. Communication challenges during the development and introduction of a new meningococcal vaccine in Africa. Clin Infect Dis. 2015 Nov 15;61 Suppl 5:S451–8. 10.1093/cid/civ493 26553674 PMC4639482

[51] Keane E, Funk AL, Shimakawa Y. Systematic review with meta-analysis: the risk of mother-to-child transmission of hepatitis B virus infection in sub-Saharan Africa. Aliment Pharmacol Ther. 2016 Nov;44(10):1005–17. 10.1111/apt.13795 27630001

[52] de Villiers MJ, Nayagam S, Hallett TB. The impact of the timely birth dose vaccine on the global elimination of hepatitis B. Nat Commun. 2021 Oct 28;12(1):6223. 10.1038/s41467-021-26475-6 34711822 PMC8553835

[53] Preventing mother-to-child transmission of hepatitis B: operational field guidelines for delivery of the birth dose of hepatitis B vaccine. Geneva: World Health Organization; 2006. Available from: https://apps.who.int/iris/handle/10665/272905. [cited 2022 May 25].

[54] Matthias DM, Robertson J, Garrison MM, Newland S, Nelson C. Freezing temperatures in the vaccine cold chain: a systematic literature review. Vaccine. 2007 May 16;25(20):3980–6. 10.1016/j.vaccine.2007.02.052 17382434

[55] Ren Q, Xiong H, Li Y, Xu R, Zhu C. Evaluation of an outside-the-cold-chain vaccine delivery strategy in remote regions of western China. Public Health Rep. 2009 Sep-Oct;124(5):745–50. 10.1177/003335490912400517 19753953 PMC2728668

[56] Nelson C, Froes P, Dyck AM, Chavarría J, Boda E, Coca A, et al. Monitoring temperatures in the vaccine cold chain in Bolivia. Vaccine. 2007 Jan 5;25(3):433–7. 10.1016/j.vaccine.2006.08.017 17000036

[57] Scott N, Palmer A, Morgan C, Lesi O, Spearman CW, Sonderup M, et al. Cost-effectiveness of the controlled temperature chain for the hepatitis B virus birth dose vaccine in various global settings: a modelling study. Lancet Glob Health. 2018 Jun;6(6):e659–67. 10.1016/S2214-109X(18)30219-5 29773122

[58] Seaman CP, Morgan C, Howell J, Xiao Y, Spearman CW, Sonderup M, et al. Use of controlled temperature chain and compact prefilled auto-disable devices to reach 2030 hepatitis B birth dose vaccination targets in LMICs: a modelling and cost-optimisation study. Lancet Glob Health. 2020 Jul;8(7):e931–41. 10.1016/S2214-109X(20)30231-X 32562649

